# Transcutaneous auricular vagus nerve stimulation improves social deficits through the inhibition of IL-17a signaling in a mouse model of autism

**DOI:** 10.3389/fpsyt.2024.1393549

**Published:** 2024-06-27

**Authors:** Wenjing Zhang, Zhiwei Mou, Qi Zhong, Xiaocao Liu, Lan Yan, Lei Gou, Zhuoming Chen, Kwok-Fai So, Li Zhang

**Affiliations:** ^1^ Department of Rehabilitation Medicine, The First Affiliated Hospital of Jinan University, Guangzhou, China; ^2^ Department of Rehabilitation Medicine, The Fifth Affiliated Hospital of Jinan University, Heyuan, China; ^3^ Lab of Regenerative Medicine in Sports Science, School of Physical Education and Sports Science, South China Normal University, Guangzhou, China; ^4^ Key Laboratory of Central Nervous System (CNS) Regeneration (Ministry of Education), Guangdong–Hong Kong–Macau Institute of Central Nervous System (CNS) Regeneration, Jinan University, Guangzhou, China

**Keywords:** autism spectrum disorder, taVNS, social ability, IL-17A, microglia

## Abstract

**Background:**

Maternal exposure to inflammation is one of the causes of autism spectrum disorder (ASD). Electrical stimulation of the vagus nerve exerts a neuroprotective effect via its anti-inflammatory action. We thus investigated whether transcutaneous auricular vagus nerve stimulation (taVNS) can enhance social abilities in a mouse model of ASD induced by maternal immune activation (MIA).

**Methods:**

ASD mouse model were constructed by intraperitoneal injection of polyinosinic:polycytidylic acid (poly (I:C)). TaVNS with different parameters were tested in ASD mouse model and in C57BL/6 mice, then various behavioral tests and biochemical analyses related to autism were conducted. ASD model mice were injected with an interleukin (IL)-17a antibody into the brain, followed by behavioral testing and biochemical analyses.

**Results:**

TaVNS reduced anxiety, improved social function, decreased the number of microglia, and inhibited M1 polarization of microglia. Additionally, taVNS attenuated the expression of the IL-17a protein in the prefrontal cortex and blood of ASD model mice. To examine the possible involvement of IL-17a in taVNS-induced neuroprotection, we injected an IL-17a antibody into the prefrontal cortex of ASD model mice and found that neutralizing IL-17a decreased the number of microglia and inhibited M1 polarization. Furthermore, neutralizing IL-17a improved social function in autism model mice.

**Conclusion:**

Our study revealed that reduced neuroinflammation is an important mechanism of taVNS-mediated social improvement and neuroprotection against autism. This effect of taVNS could be attributed to the inhibition of the IL-17a pathway.

## 1 Introduction

Autism is a neurodevelopmental disorder characterized by social impairment and stereotyped behavior. As there is no specific treatment currently, autism spectrum disorder imposes an enormous burden on patients, families and human society. Neuroimmune mechanisms may play an important role in autism (1). Immune cells such as microglia and T lymphocytes in the brain are crucial for normal brain development and function. Microglia are involved in regulating the programmed death of neurons, promoting synaptogenesis, and integrating functional neuronal circuits. Furthermore, while T helper regulatory cells (Tregs) promote myelination (2), T helper 17 (Th17) cells promote demyelination (3). Therefore, abnormalities in microglia and several types of immune cells may also contribute to abnormalities in brain development. Microglial dysfunction is associated with core ASD symptoms, such as stereotypic behavior (4), and there is a positive correlation between the severity of ASD symptoms and the levels of Th17 cells and/or related cytokines, such as IL-17a (5, 6). Studies have shown that IL-17a enhanced both M1 and macrophage-polarizing signals *in vivo* and *in vitro* (7).

MIA is a major environmental factor capable of increasing the incidence of autism and schizophrenia, MIA affects fetal neurodevelopment by activating maternal inflammatory pathways during pregnancy and increasing the levels of inflammatory factors that cross the placenta and blood-brain barrier (8). Lipopolysaccharide (LPS), a component of the bacterial cell wall, or polyinosinosine polycytilic acid (poly I:C), a synthetic double-stranded RNA (dsRNA) analog, are commonly used to elicit bacterial- or viral-like innate immune responses (9). Studies have shown that Poly I:C-induced MIA offspring exhibit increased repetitive behaviors and social behavior deficits that are representative of ASD (10). Earlier studies have shown that the offspring of pregnant mice infected with the virus or injected intraperitoneally with poly(I:C) exhibit behavioral symptoms similar to those of ASD: social deficits, abnormal communication, and repetitive behaviors (11). Over the past 15 years, poly I:C-induced MIA models have been frequently used as preclinical models to study changes associated with ASD in rodent neural circuits and associated behavioral phenotypes (12–16), as evidenced by the extensive literature, and this viral MIA model has shown substantial face, structure, and predictive validity (9). Therefore, we used MIA-induced ASD mouse model for experiments.

VNS can be used in treating epilepsy (17), treatment-resistant depression (18), sepsis (19), stroke (20), traumatic brain injury (21), cardiovascular control (22), and pain management (23). In one original investigation, VNS therapy was shown to be safe and effective for treating focal, generalized and combined epilepsy types (24), possibly because VNS is involved in reducing the excitability of the limbic system, thalamus, and corticotropic areas; and regulating neural circuits in the brainstem, midbrain, and cortex (25). The two-year response rate to VNS in patients with treatment-resistant depression ranged from 42% to 53.1% (≥50% reduction in the Hamilton Depression Scale score from baseline) (26), and the mechanism is speculated to the modulation of cytokines and the regulation of microglial activation to attenuate the inflammatory response by VNS (27). In addition, VNS can improve upper limb dysfunction, dysphagia, and cognitive dysfunction in stroke patients, and Zhao reported that VNS may promote microglial M2 polarization by inhibiting IL-17a and thus prevent cerebral ischemia/reperfusion injury (28). Therefore, we hypothesized that taVNS affects microglial number and M1 polarization in an IL-17a-dependent manner. In the present study, we explored the effects of taVNS on IL-17a expression and neuroinflammation in a mouse model of autism induced by MIA, as well as its effect on the social behavior of ASD model mice.

## 2 Materials and methods

### 2.1 Animals

The male and female C57BL/6 mice (8 to 10 weeks old, 23–25 g) used in the study were purchased from Guangdong Experimental Animal Centre and housed in a standard environment (room temperature controlled at 22 ± 2°C, 12-h light-dark cycle) with food and water available ad libitum. The experimental protocol was approved by the First Affiliated Hospital of JiNan University Ethics Committee and animal experiments. Efforts were made to minimize animal suffering during the experiments and the number of animals used in the research.

### 2.2 Maternal immune activation induces an ASD-like mouse model

Mice were mated overnight, and the presence of a vaginal plug, indicating successful mating, was observed; these mice were designated embryonic day 0.5 (E0.5). The pregnant dams were weighed and administered 20 mg/kg poly (I:C) (Millipore Sigma) or saline by i.p. injection on E12.5 (29). As part of the MIA treatment, the MIA offspring were weaned from their mothers on postnatal day 21 and housed with same-sex littermates, with three to five mice per cage. MIA offspring with social impairment were considered as exhibiting autism-like behaviors. One male and one female offspring was selected from each litter to minimize litter effects.

### 2.3 Transcutaneous auricular vagus nerve stimulation

After intraperitoneal injection of 1.25% tribromoethanol anesthesia, the mice were placed on a surgical towel with a heating pad to expose the left ear, and the special electrode was fixed on the stimulation site of the left ear of the mice after wiping with 72% alcohol. In the real stimulation group, special electrodes were placed in the upper and lower auricular branch of the vagus nerve (ABVN) innervated areas of the mouse tragus for electrical stimulation intervention. The electrical stimulation parameters were as follows:1) 0.5 mA, 20 Hz, 30 s ON and 5 min OFF, with a total length of 30 minutes and a pulse width of 500 μs, the mice were recorded as the taVNS-1 group; 2) 1 mA, 20 Hz, 30 s ON and 5 min OFF, with a total length of 30 minutes and a pulse width of 330 μs, the mice were recorded as the taVNS-2 group. In the sham stimulation group, a series of similar stimulations were performed by placing the electrodes outside the innervation area of the ABVN of the mouse ear margin. After stimulation, the mouse was returned to the cage and waited for it to wake up. Each mouse was stimulated for 30 min, and the intervention was performed once a day from 9:00 to 10:00 in the morning for a total of 7 days. Each group of mice was stimulated with real stimulation and sham stimulation at 6w after birth.

### 2.4 Stereotaxic injection and IL-17a blockade

The mice were anaesthetized via intraperitoneal injection of 1.25% tribromoethanol at 6w age. The anaesthetized mouse brain was fixed on a stereotaxic instrument, the top of the head was routinely skin-prepared and disinfected, the top of the head was cut approximately 1.5 cm in the mid-sagittal direction, and the bregma was clearly exposed by wiping with 75% alcohol cotton balls. Positioning was performed according to the stereotaxic atlas. After confirming that the anterior and posterior fontanelles were on the same horizontal line, the anterior bregma was taken as the zero point, and the following injection sites were selected: mPFC (AP2.0 mm, ML+0.2 mm, DV-2.0 mm) (30). Four microliters of sterile cerebrospinal fluid (the ingredient ratios are shown in **Table 1**) or IL-17A antibody were drawn up with a 5 µl microsyringe and injected at a rate of 0.42 µl/min. After the injection, the needle was left in place for 10 minutes, after which the needle was slowly withdrawn to prevent the drug from flowing back into the brain along the needle track. After the injection, the skin was sutured, the wound was disinfected again to prevent infection, and the mouse was subsequently placed on the inside of an empty cage to allow it to rest until it awoke. At this time, attention should be given to keeping warm. Control group mice were injected sterile cerebrospinal fluid; MIA mice were randomly divided into two groups, one group was the ASD group, all mice in the ASD group were injected with sterile cerebrospinal fluid and did not block IL-17a, which was considered to be a sham injection control group; another group was the anti-IL-17a group, all of whom were injected with IL-17a antibodies, and all mice in the anti-IL-17a group were blocked from IL-17a in the brain.

**Table 1:** Sterile cerebrospinal fluid composition ratio.

Ingredients	Concentration(nmol/L)	Corporation	Molecular mass
NaCl	126	Sigma	58.44
KCl	2.5	Sigma	74.56
Glucose	10	Sigma	180.16
NaH_2_PO_4_	1.25	Sigma	120
NaHCO_3_	26	Sigma	84.1
MgCl_2_	2	Sigma	95.2
CaCl_2_	2	Sigma	147.02

### 2.5 Open field experiment

The open field test site was an acrylic box with a size of 50 cm × 50 cm × 50 cm, and the site was kept clean and dry. The 30 cm× 30 cm range in the center of the field was set as the central area. The mouse was gently placed in the center of the field, and the camera and timing were performed at the same time. After 5 min of detection, the camera was stopped. During this period, the mouse could move freely in the field, and EthoVision software was used to record the movement route and related data of the mouse within 5 minutes, including the total distance the mouse moved in the field and the time the mouse stayed in the central area of the field. Before and after each mouse test, the inner wall and bottom of the square box were wiped with 75% alcohol to eliminate the information left in the box when the previous mouse was tested (such as the smell of urine and urine of the mouse) to avoid affecting the test results of the next mouse. After the experiment, the total distance that each mouse moved in the field and the time that the mouse stayed in the central area of the field were recorded. OPF was performed first at 6 w after the birth of mice, and another OPF was performed after the taVNS intervention ended.

### 2.6 Three-chamber social preference test

The three-chamber sociability test box was an acrylic box with dimensions of 60 cm × 40 cm × 20 cm, which was kept clean and dry. The box was separated into translucent plastic plates and divided into three chambers (20 cm × 40 cm × 20 cm in size), and there was a 6 cm gap in the middle of the partitions for the mice to pass through freely. Before the start of the test, the tested mice were individually placed in the center of the test field for free adaptation for 3 min. Mice without position preference in the adaptation phase were used for the next test. In three stages, the animals were tested under dark light, and each stage lasted 10 min. In the first stage, two empty cylindrical metal cages (9 cm × 15 cm) were placed on both sides of the three chambers, and the mice were allowed to explore freely in the field for 10 min. In the second stage, tool mice of the same sex (the same strain was never contacted before, 6 weeks old) were randomly placed on one side of three empty cylindrical metal cages on both sides. The side of the cage with tool mice was defined as the social area, the middle chamber was defined as the middle area, and the side with the empty cage was defined as the blank area. The mice were allowed to explore the field freely for 10 min. The social preference index in the second stage was calculated as follows: (total exploration time in the social area - total exploration time in the blank area)/(total exploration time in the social area + total exploration time in the blank area) × 100%. In the third stage, another tool mouse of the same sex (same strain, never contacted before, 6 weeks old) was placed in an empty cylindrical metal cage on the other side of the three chambers. The side of the mouse cage with the new tools was defined as the new social area, the middle chamber was defined as the middle area, and the side of the mouse cage with the old tools was defined as the old social area. The social preference index of the third stage = (total exploration time in the new social area - total exploration time in the old social area)/(total exploration time in the new social area + total exploration time in the old social area) × 100%. Before and after the test, each chamber and cage were wiped with 75% alcohol to remove the smell, and the next mouse was tested after the alcohol evaporated. Three-chamber social preference test was performed at 6w after the birth of mice, and another three-chamber social preference test was performed after the taVNS intervention. The DI of cage means the sniffing time of the mouse against the social mouse, a higher value of DI indicates that the tested mouse will sniff the new social mouse for longer than the empty cage or the old social mouse. The DI of chamber means the time that the tested mouse is in the chamber where the social mouse is, and the higher the value, the longer the mouse is in the chamber of the new social mouse relative to the chamber of the empty cage or the old social mouse.

### 2.7 Immunohistochemistry

After the final behavioral behavioral test, the mice were anaesthetized with 1.25% tribromoethanol intraperitoneally, and the thorax was cut upwards along the abdominal cavity to expose the heart. A perfusion needle was inserted into the left ventricle, the right atrial appendage was cut open, and a rapid perfusion of ice saline was used through the heart to flush the blood from all over the body. After the liver is evenly whitened, the 4% paraformaldehyde solution (PFA) was replaced and perfused until the mice were completely stiffed. Then, the brain was removed, fixed overnight in 4% PFA, dehydrated in 10%, 20%, and 30% sucrose solution for 2 h and 24 h, and allowed to sank to the bottom. The dehydrated brain tissue was fixed on a frozen stage with 30% sucrose solution and cut into 4 μm thick coronal sections with a cryostat. After the cells were incubated with blocking solution (9:1 ratio of blocking solution to 3% Triton X-100) for 2 hours, primary antibodies against Iba-1 (1:300; Wako, 019–19741) or CD16 (1:300; R&D Systems, AF1460) were used for staining overnight. After rinsing, the sections were incubated with Alexa Fluor 488 (1:500; Abcam, ab150077) or Alexa Fluor 488 (1:500; Invitrogen, A11015) for 2 hours. After washing, the number of immunostained cells was counted in representative fields of the ipsilateral hemisphere by two independent observers blinded to the experiment.

### 2.8 Western blot

After the final behavioral test, the mice were sacrificed by cervical dislocation, the brain tissue was quickly removed, and the total protein was extracted using a commercially available kit (Beyotime, China). Equal amounts of protein samples (20 μg) were separated on a 12% gel, electrophoretically transferred to PVDF membranes and detected with enhanced chemiluminescent Western blot detection reagents (Thermo Scientific, USA). The following primary antibodies were used in the study: anti-IL-17A (1:5000; Abcam; ab79056), anti-TNF-α (1:1000; Abcam; 183218), anti-GAPDH (1:1000; Cell Signaling; 5174S), anti-Tublin (1:1000; Cell Signaling; 2148S), Goat Anti-Rabbit IgG H&L (HRP) (1:5000; Abcam; ab6721). Protein bands were visualized using an imaging system (Bio-Rad, USA). Integrated gray values of each band were measured using ImageJ (National Institutes of Health, Bethesda, USA).

### 2.9 ELISA

After the final behavioral test, the mice were anaesthetized, the eyeballs of the mice were removed to obtain venous blood, and the blood was centrifuged for serum. Then the mice were sacrificed by cervical dislocation, and the mPFC was taken, triturated and centrifuged to obtain supernatant. The quantification of IL-1β, IL-2, IL-10, and IL-17a was performed in mPFC and serum samples using ELISA kits (Jianglaibio, China) following the manufacturer’s instructions. The optical density (OD) of each sample was subsequently measured at a wavelength of 450 nm using a microplate reader.

### 2.10 Statistical analysis

In this experiment, GraphPad Prism 8 software was used for recording and analysis. The D’Agostino & Pearson test and Shapiro-Wilk test were used to check the data distribution. One-way repeated-measures ANOVA was then used, followed by the Holm-Sidak multiple comparisons test. If the data do not conform to the normal distribution, the Kruskal-wallis H test is used. *P* < 0.05 was considered to represent statistical significance.

## 3 Results

### 3.1 taVNS ameliorates anxiety and social deficits in poly(I:C)-induced ASD model mice

**Figure 1:**
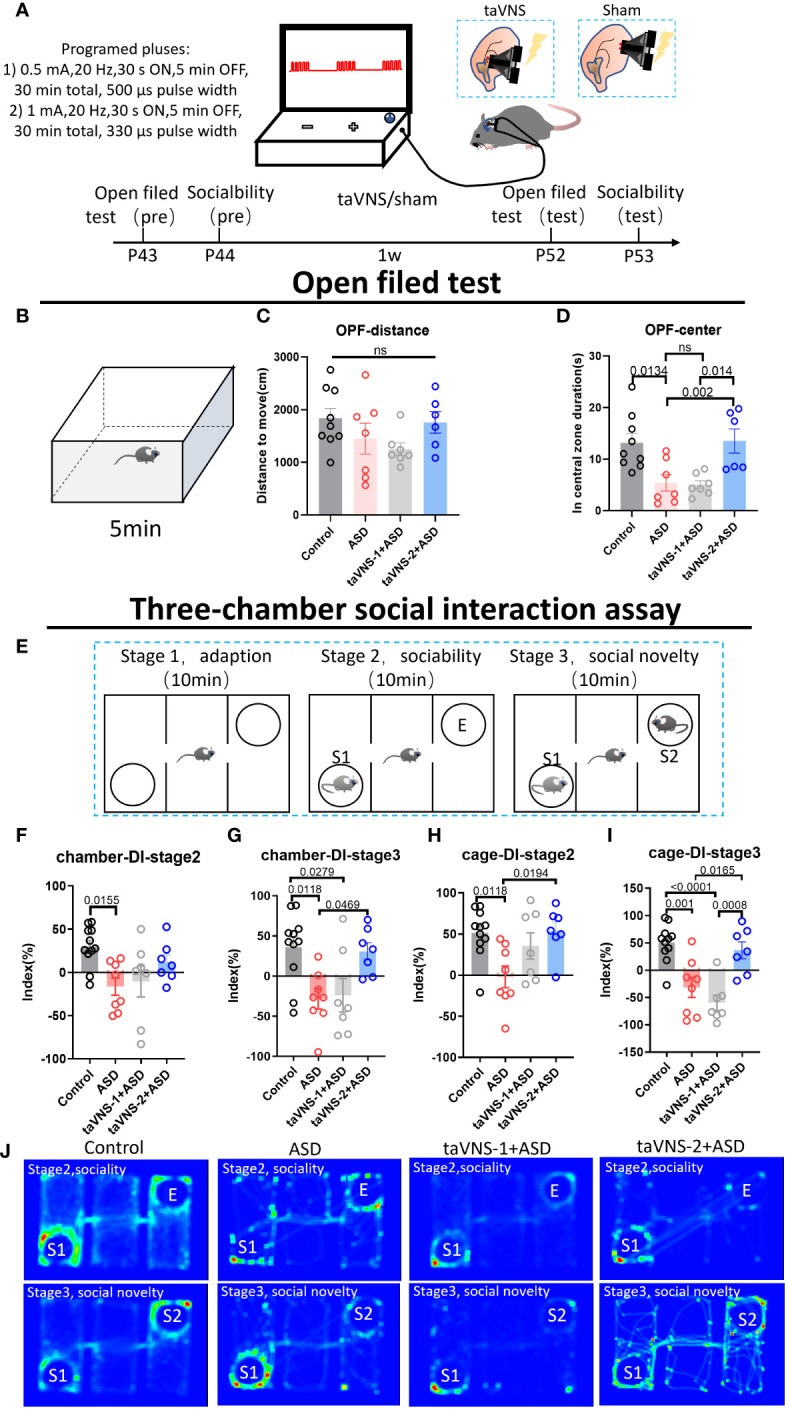
TaVNS improves anxiety and sociability deficits in poly(I:C)-induced ASD model mice. **(A)** Schematic diagram of the experiment. Behavioral tasks were performed at 6 w, and taVNS-1/2 or sham stimulus intervention was performed daily for 7 days; subsequently, the behavioral tasks were performed. **(B)** Schematic diagram of the open field test in mice. Mice in each group were tested in the open field for 5 min. **(C, D)** Results of the open field test after intervention. Statistical results showed significant changes in the time spent in the center, and no significant difference in the distance (n= 9, 7, 7, and 6). **(E)** Schematic diagram of a three-chamber social experiment. Mice were tested for 10 min each in three stages. **(F-I)** Results of the three-chamber sociability test after intervention. Statistical results showed significant differences in the exploration time to new mice (n=11, 7, 7, and 7). **(J)** Heatmap of the three-chamber social experiment in mice.

To optimize the ability of the taVNS parameters to improve social behavior, we employed the open field test and three-chamber sociability test taVNS intervention in autism model mice (**Figure 1A**). Our results revealed no significant difference in total distance travelled in the open field among the four groups. However, compared with the control group and the taVNS-2 group, the stay time of mice in the central area of the open field was significantly lower. Besides, compared with the ASD group and the taVNS-1 group, the mice in the taVNS-2 group stayed longer in the central area of the open field. These findings indicate that only high-power taVNS-2 alleviated the anxiety-like behaviors of the ASD model mice (**Figures 1B–D**, *P* < 0.05). The results of the subsequent three-chamber sociability test also showed similar results. In the second stage, the ASD model mice showed lower social willingness than the control group mice, and the sniffing time on the new tool mice and time spent in the same chamber with the tool mice of the ASD model mice were significantly lower than those of the control mice group. The time for taVNS-2 group mice to sniff tool mice was significantly higher than that of the ASD group. In the third stage of the test, ASD model mice also showed lower social willingness than the control group mice, while compared with the ASD group, the sniffing time on the new tool mice and the time in the same chamber with the new tool mice were significantly higher in mice after the high-power taVNS-2 intervention. But the mice in low-power taVNS-1 group did not show the same phenomenon, which suggested among those whose social preference indices were significantly lower in the ASD group, only the taVNS-2 group showed improvements in social dysfunction (**Figures 1E–J**, *P* < 0.05). Therefore, the stimulation parameters of taVNS-2 will be used for subsequent experimental interventions.

### 3.2 taVNS modulates immune responses by targeting IL-17a in the prefrontal cortex

**Figure 2:**
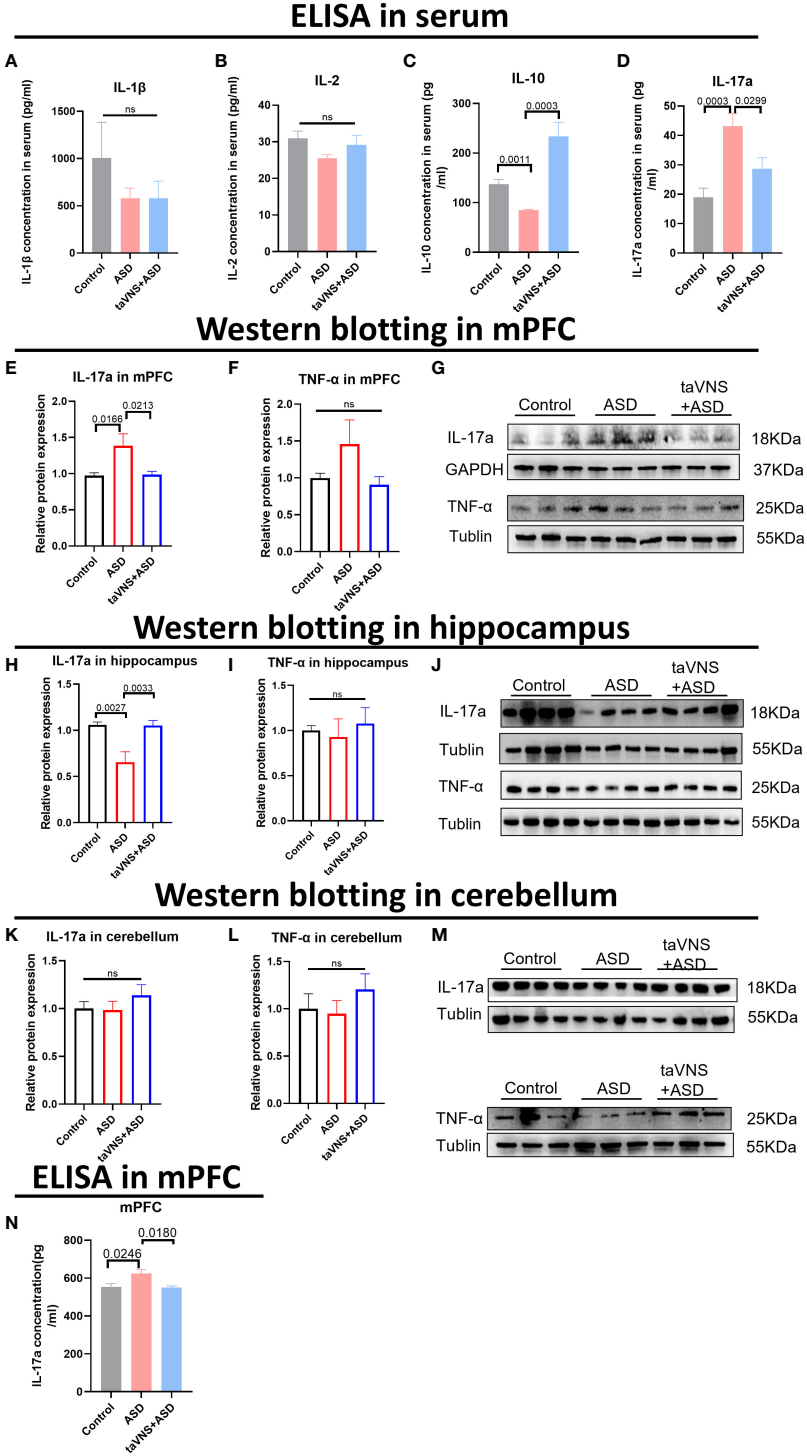
TaVNS preferentially attenuated the increase in IL-17a levels in the mPFC area. **(A-D)** ELISA was used to detect the expression levels of IL-1β, IL-2, IL-10, and IL-17a in the serum. Statistical results showed significant changes in IL-10 and IL-17a, and no significant difference in IL-1β and IL-2 (IL-1β, n=5,7,7; IL-2, n=11,5,5; IL-10, n=11,3,5; and IL-17a, n=12,12,12). **(E, F)** WB detection of the relative expression levels and bands corresponding to IL-17a and TNF-α in the mPFC. Statistical results showed significant changes in IL-17a, and no significant difference in TNF-α (IL-17a, n=14, 14, and 14; TNF-α, n=7, 6, and 7). **(G)** Protein blotting bands for IL-17a and TNF-α in the mPFC. **(H, I)** WB analysis of the relative expression levels and bands corresponding to IL-17a and TNF-α in the hippocampus. Statistical results showed significant changes in IL-17a, and no significant difference in TNF-α (8 IL-17a-treated mice per group and 7 TNF-α-treated mice per group). **(J)** Protein blotting bands for IL-17a and TNF-α in the hippocampus. **(K, L)** WB analysis of the relative expression levels and bands corresponding to IL-17a and TNF-α in the cerebellar region. Statistical results showed no significant change in IL-17a and TNF-α (8 IL-17a-treated mice per group and 7 TNF-α-treated mice per group). **(M)** Protein blotting bands for IL-17a and TNF-α in the cerebellar region. **(N)** ELISA detection of IL-17a levels in the mPFC. Statistical results showed significant changes in IL-17a (n=5 in control group, ASD group, and taVNS group).

To evaluate the effect of taVNS on the expression of inflammatory factors in the serum of ASD model mice, we measured the levels of IL-1β, IL-2, IL-10, and IL-17a and found that taVNS reduced the level of IL-17a in the serum of ASD model mice while increasing the expression of IL-10 (**Figures 2A–D**, *P* < 0.05). Subsequently, we used Western blotting to measure the protein levels of IL-17a in different brain regions. The results showed that there was significantly greater IL-17a expression in the prefrontal cortex of the ASD group than in the control group, and taVNS treatment decreased the IL-17a level (**Figures 2E–G**, *P* < 0.05). The opposite trend was observed in the hippocampus (**Figures 2H–J**, *P* < 0.05), but no significant change was observed in the cerebellar region (**Figure 2K-M**, *P* > 0.05). The ELISA results for the mPFC also supported the elevation of IL-17a in the ASD group (**Figure 2N**, *P* < 0.05).

### 3.3 Treatment with taVNS modulates the number and polarization of microglia

**Figure 3:**
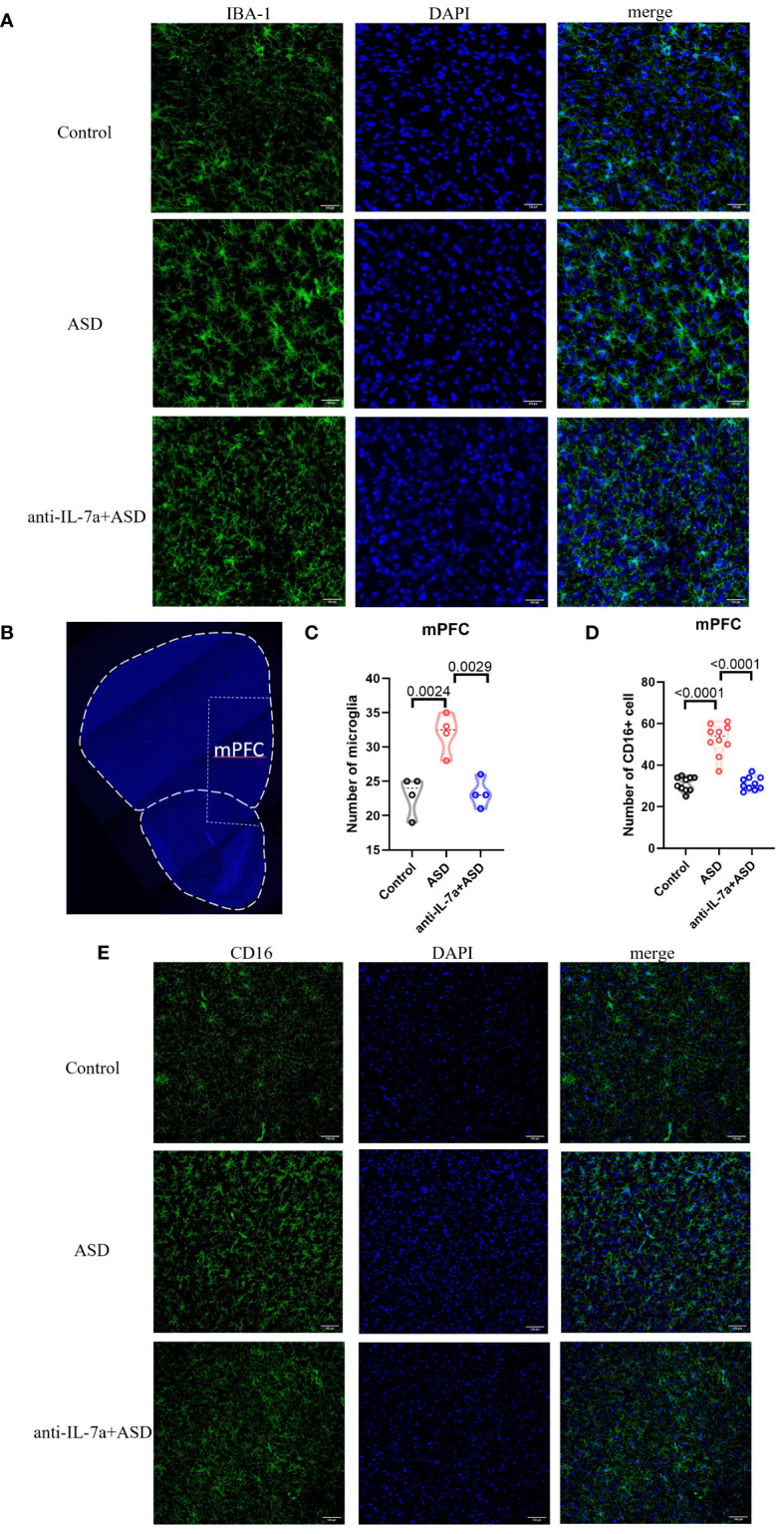
TaVNS intervention reduces the number of microglia and M1 polarization. **(A)** Immunofluorescence staining of IBA-1 and DAPI in the prefrontal cortex (scale bars, 100 μm). **(B)** Coronal section displaying the mPFC (scale bars, 30 μm). **(C)** Count of IBA-1^+^ cells in the mPFC. Statistical results showed significant changes in IBA-1^+^ cells (n=4 in each group). **(D)** The number of CD16^+^ cells in the mPFC. Statistical results showed significant changes in CD16^+^ cells (n=10 in each group). **(E)** Immunofluorescence staining of CD16 and DAPI in the prefrontal cortex (scale bars, 100 μm).

We next examined the effect of taVNS intervention on the number and polarization of microglia in the mPFC area in MIA-induced ASD model mice by immunofluorescence staining. The results showed a significantly greater number of IBA-1+ microglia in the ASD group than in the control group, while the taVNS group had fewer dense IBA-1+ cells than the ASD group (**Figures 3A–C**, *P* < 0.05). Next, we investigated the effect of taVNS on the polarization of microglia in the mPFC. Immunofluorescence imaging revealed a significantly greater number of M1 microglia expressing CD16 in the ASD group than in the control group, and the taVNS group had a lower number of M1 microglia expressing CD16 (**Figures 3B, D, E** *P* < 0.05).

### 3.4 Prefrontal cortical inhibition of IL-17a recapitulates the effect of taVNS on improved sociability

**Figure 4:**
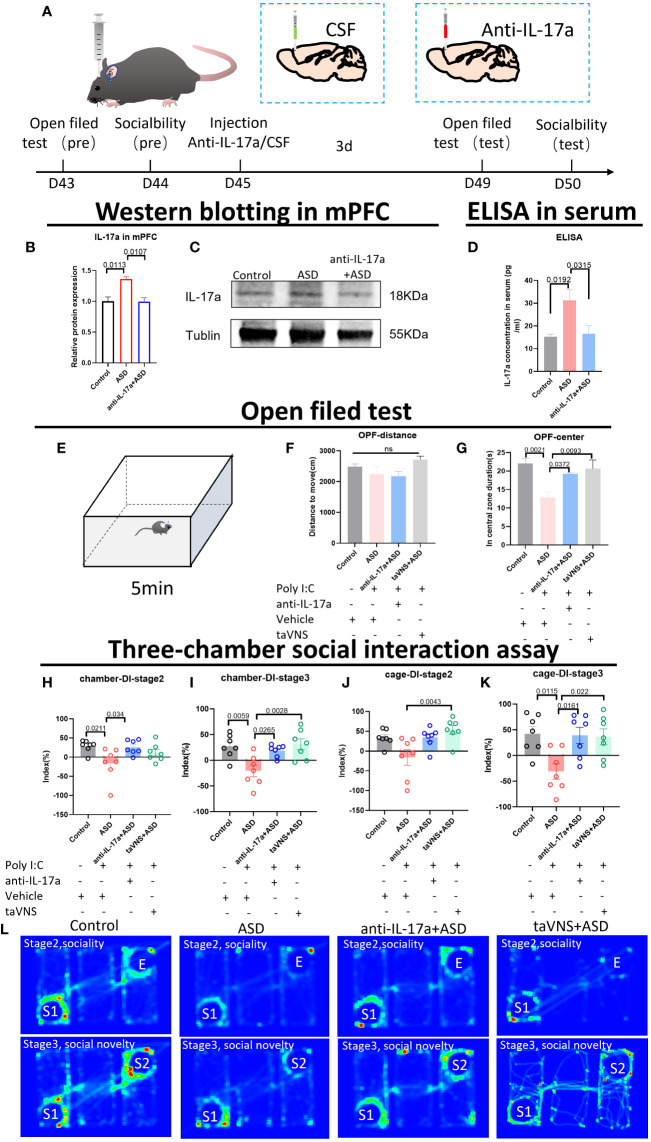
Reducing the level of IL-17a improves the anxiety state and social function of autism model mice. **(A)** Schematic diagram of the experiment. The animals were subjected to behavioral tests at 6 weeks. Anti-IL-17a antibody or sterile cerebrospinal fluid was injected into the prefrontal cortex, and the mice were subjected to behavioral tasks 3 days later. **(B)** WB detection of the relative expression levels and bands corresponding to IL-17a in the mPFC. Statistical results showed significant changes in IL-17a (n=3 in each group). **(C)** Protein blotting bands for IL-17a and TNF-α in the cerebellar region. **(D)** ELISA detection of IL-17a levels in serum. Statistical results showed significant changes in IL-17a (n=5 in each group). **(E)** Schematic diagram of the open field test in mice. Mice in each group were tested in the open field for 5 min. **(F, G)** Results of the open field test after intervention. Statistical results showed significant changes in the time spent in the center, and no significant difference in the distance (n=7 in each group). **(H-K)** Results of the three-chamber sociability test after intervention (n=7 in each group). **(L)** Heatmap of the three-chamber social experiment in mice.

To verify whether IL-17 is the key target through which taVNS improves social function in ASD model mice, we injected an IL-17a antibody into the prefrontal cortex of mice. Western blotting and ELISA revealed that the IL-17a concentration in the mPFC and serum was significantly lower in the antibody-injected group than in the ASD group (**Figures 4A–D**, *P* < 0.05). The behavioral results showed that the inhibition of IL-17a in the prefrontal cortex reproduced the effect of taVNS on improving social ability. There was no significant difference in the total distance travelled in the open field among the four groups, but the time spent in the central area was significantly greater in the anti-IL-17a group than that in ASD group (**Figures 4E–G**, *P* < 0.01). A subsequent three-chamber sociability test also showed that the social preference index was significantly greater in the anti-IL-17a group than that in ASD group (**Figures 4H–L**, *P* < 0.05). In summary, neutralizing IL-17a alleviated the anxiety of ASD model mice and improved social deficits.

### 3.5 IL-17a blockade rescues microglial abnormalities in MIA offspring

**Figure 5:**
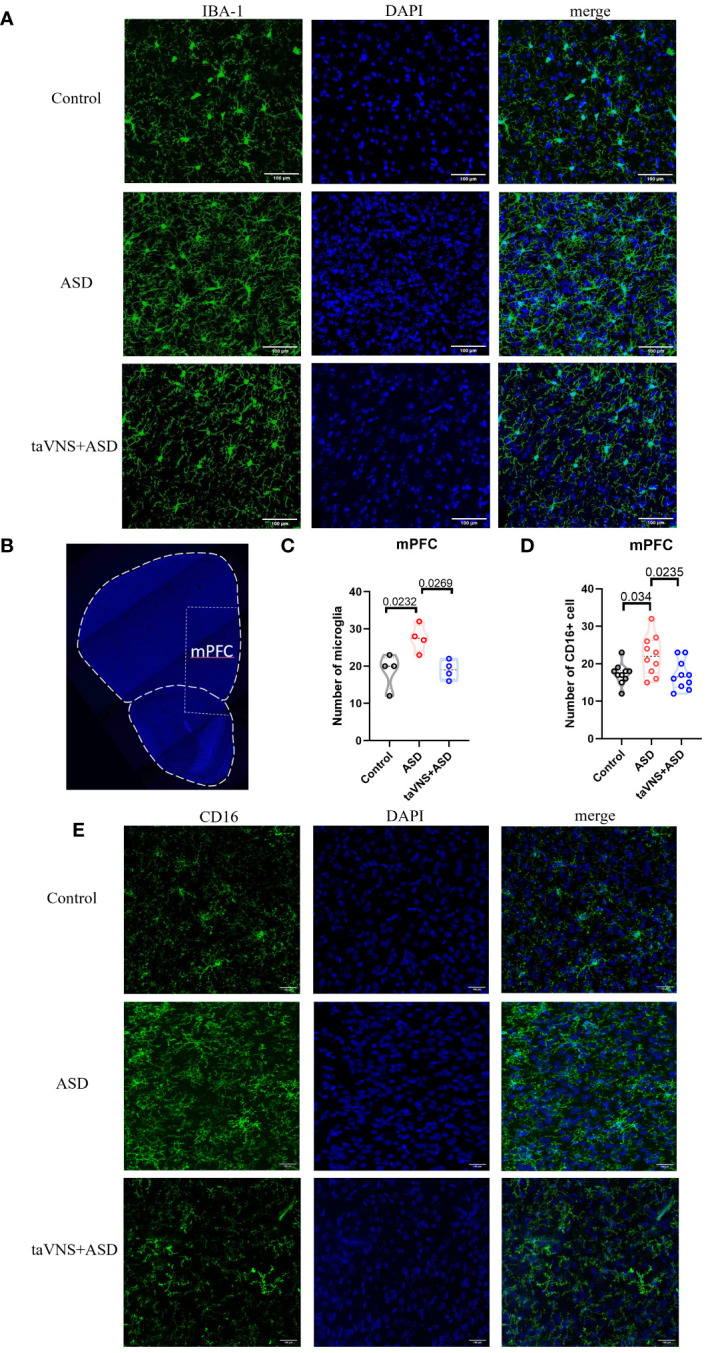
IL-17a blockade rescues microglial abnormalities in MIA offspring. **(A)** Immunofluorescence staining of IBA-1 and DAPI in the mPFC (scale bars, 100 μm). **(B)** A coronal section of the mPFC (scale bars, 30 μm). **(C)** Quantification of IBA-1^+^ cells in the prefrontal cortex. Statistical results showed significant changes in IBA-1^+^ cells (n=4 in each group). **(D)** CD16^+^ cell count in the prefrontal cortex. Statistical results showed significant changes in CD16^+^ cell (n=10 in each group). **(E)** Immunofluorescence staining of CD16 and DAPI in the prefrontal cortex (scale bars, 100 μm).

We quantified the number of microglia in the mPFC, and the results showed that the number of microglia was significantly reduced after neutralization treatment (**Figures 5A–C**, *P* < 0.05). To evaluate the effect of neutralizing IL-17a on microglial polarization, we performed immunofluorescence staining and found that there was a statistically significant increase in the number of CD16-expressing M1 microglia in the ASD group compared to that in the control group (**Figures 5B, D, E**; *P* < 0.05). The number of CD16-expressing M1 microglia in the anti-IL-17a group was significantly lower than that in the ASD group (**Figures 5B, D, E**; *P* < 0.05).

## 4 Discussion

Autism is a neurodevelopmental disorder characterized by social impairment and stereotyped behavior. Since the incidence rate of autism is increasing year by year, it is urgent to find a therapy for autism. Studies of various brain disorders have shown that vagus nerve stimulation (VNS) exerts neuroprotective effects via anti-inflammatory effects (25, 31). Although the exact anti-inflammatory mechanism of VNS is not fully understood, it may be related to the regulation of cytokine release in peripheral immune cells, the state of microglia, and changes in the permeability of the blood-brain barrier (BBB) (32). Many autistic patients exhibit parasympathetic nervous system dysregulation which is often accompanied by decreased vagal tone, and reduced vagal activity is associated with autistic behavior and language impairment (33), while increased vagal activity sometimes predicts a more favorable prognosis (34). VNS may ameliorate neurodevelopmental impairments through altered parasympathetic activity (35). Studies using VNS in children with epilepsy and autism spectrum disorder have generated some positive results, as VNS reduces seizure frequency and improves quality of life in ASD patients (36–39). A study of remotely supervised at-home taVNS for the treatment of ASD demonstrated improvement in anxiety and drowsiness after 2 weeks of taVNS intervention, demonstrating that remote at-home taVNS intervention is feasible and effective (38). In the largest study to date on VNS treatment for ASD patients, patients had remarkable seizure reductions (39). After 12 months of VNS treatment, 56% of the nonautistic patients and 62% of the autistic patients experienced a greater than 50% reduction in the frequency of seizures. Individuals also showed improvements in alertness, verbal communication, memory, and academic/career achievement. However, compared with people without autism, ASD patients also experienced significant improvements in mood after 12 months of VNS treatment (39). As shown in the pilot taVNS study in ASD patients mentioned above, the impact of VNS on social function in patients with autism has not been investigated.

Social behavior is mediated by a massively distributed brain network consisting of multiple brain structures. Current studies have shown that mPFC, hippocampus, and cerebellum are all involved in the regulation of social behavior. Neurons in mPFC form communication networks with various subcortical regions, such as the nucleus accumbens (NAc), the amygdala, the ventral tegmental area (VTA), and the occipital nucleus (40, 41), and play an important role in regulating cognition, mood, and behavior (42, 43). When mice approach strangers, the firing rate of some mPFC neurons increases (44), while optogenetically induced sustained elevation of mPFC pyramidal neuronal excitability decreases social interaction behavior (45). mPFC is also involved in the adverse effects of social isolation stress. The activity of mPFC neurons projected to the posterior paraventricular thalamus (pPVT) is enhanced during social interaction, and inhibition of this projection decreases social ability (46). In addition, the hippocampus is also a key structure involved in social interaction, and it is composed of three cornu ammonis (CA) subregions, including CA1, CA2, and CA3 (47). In particular, the CA2 brain region, which is an area involved in social behavior, may mediate social recognition and play an important role in social memory formation (48). Gene-targeted inactivation of CA2 pyramidal neurons results in a marked loss of social memory, i.e., the ability of animals to remember their own animals without changes in social competence or several other hippocampus-dependent behaviors, including spatial and contextual memory, and these behavioral and anatomical results reveal that CA2 is a key hub in social cognitive memory processing (49). Compared with the above two brain regions, the study of cerebellum in social interaction is still in the early stage, and in recent years, experiments on cerebellum and social interaction have begun to appear (50). Researchers in the field of social neuroscience have discovered that the important social function of the cerebellum, that is, the support of the cerebellum is needed to infer the mental state of others to reconstruct the sequence of social actions by knowing the correct sequence of their behavior (51). Therefore, we targeted these three socially relevant brain regions.

Given that social barriers are one of the prominent functional impairment in autism, we used taVNS with different parameters to intervene in ASD model mice and detected changes in social function. Results reveal that high parameter of taVNS (1 mA, 20 Hz, 30 s ON and 5 min OFF, with a total length of 30 minutes and a pulse width of 330 μs) can effectively improve anxiety and social function in ASD model mice. We also found that taVNS can significantly reduce IL-17a in mPFC and in the blood of ASD model mice. But the influence of other interleukins in the blood, such as IL-1 β, IL-2, IL-10; and TNF-α in the hippocampus and cerebellum is not significant. Furthermore, taVNS significantly reduces the number of microglia and also inhibits M1 polarization of microglia. Whether the change of IL-17a is a key factor for taVNS to play a role in improving the social function of autism model mice is worth further exploration.

Studies have shown that IL-17a can improve behavioral deficits in experimental models of autism, and immune cells and derived factors in the brain play important roles in improving autism symptoms (52). Xie et al. found that children with autism exhibit increased blood TNF-α concentrations related to symptom severity, as well as decreased expression of the THRIL gene involved in regulating TNF-α, so we also measured the expression level of TNF-α (53). A study revealed that the serum level of IL-17a was significantly increased in children with autism (5), and genome-wide copy number variation (CNV) analysis confirmed that IL17A is one of the many genes enriched in autism patients (54). IL-17a is highly conserved during the evolution of the vertebrate immune system and plays an important role in infection and autoimmune diseases. In addition, studies have shown a correlation between IL-17a and polarization of microglia, and glial cells also play an important role in the pathophysiology of autism. In ASD, changes in the number of neurons and glial cells disrupt neural circuits to affect behavior. Recent findings suggest that reactive glial cells lead to defects in synaptic function and induce autism under inflammatory conditions (55). A study also suggested that disturbances or changes in microglial physiology and defense functions, such as the failure to eliminate synapses or abnormal microglial activation, may be critical for the induction of brain diseases, including autism (56). Reactive microglia were found in the cerebellum, white matter and cortex of patients with autism. In addition to the increased density throughout the brain of ASD patients, the morphology of microglia also exhibits abnormalities, including enlarged cell bodies, as well as branch retraction and thickening (57, 58). In order to verify the effect of IL-17a on taVNS in improving ASD socialization, we conducted corresponding experiments. It is shown that after injecting IL-17a antibody into the mPFC of ASD model mice to neutralize IL-17a, the ASD mouse model reproduced the same therapeutic effect as using taVNS, which includes improved anxiety and social function, reduced number of microglia, and inhibited M1 polarization.

Unfortunately, due to the heterogeneity of ASD, a treatment may not have a beneficial effect on all types of ASD, and the study in this article can only show that taVNS has a significant effect on the MIA model, but the effect of this intervention on ASD caused by other causes is still unclear and further research is needed. Besides, taVNS can reduce IL-17a levels and improve the social function of maternal immune-activated mice, but the specific mechanism of how inflammatory factors lead to behavioral improvement was not elaborated, and subsequent experiments will be conducted to investigate this aspect.

## 5 Conclusion

Taken together, our findings suggest that reduced microglial numbers and inhibition of M1 polarization may be an important mechanisms of taVNS-mediated neuroprotection against autism. This effect was at least partially attributable to the inhibition of IL-17a expression by taVNS.

## Data availability statement

The original contributions presented in the study are included in the article/supplementary materials, further inquiries can be directed to the corresponding author/s.

## Ethics statement

The animal study was approved by the First Affiliated Hospital of Jinan University Ethics Committee and animal experiments. The study was conducted in accordance with the local legislation and institutional requirements.

## Author contributions

WZ: Data curation, Formal analysis, Methodology, Resources, Validation, Writing – original draft, Writing – review & editing. ZM: Project administration, Supervision, Writing – review & editing. QZ: Data curation, Validation, Writing – review & editing. XL: Writing – review & editing. LY: Data curation, Methodology, Writing – review & editing. LG: Investigation, Writing – review & editing. ZC: Funding acquisition, Writing – review & editing. K-FS: Writing – review & editing, Funding acquisition. LZ: Writing – review & editing, Data curation, Formal analysis, Validation.
